# Directing the Self-assembly of Tumour Spheroids by Bioprinting Cellular Heterogeneous Models within Alginate/Gelatin Hydrogels

**DOI:** 10.1038/s41598-017-04691-9

**Published:** 2017-07-04

**Authors:** Tao Jiang, Jose G. Munguia-Lopez, Salvador Flores-Torres, Joel Grant, Sanahan Vijayakumar, Antonio De Leon-Rodriguez, Joseph M. Kinsella

**Affiliations:** 1 Department of Mechanical Engineering, McGill University, Montreal, Quebec, H3A 0C3 Canada; 2 Department of Molecular Biology, Instituto Potosino de Investigación Científica y Tecnológica, A.C. (IPICyT), San Luis Potosi, San Luis Potosi, 78216 Mexico; 3 Department of Mining and Materials Engineering, McGill University, Montreal, Quebec, H3A 0C5 Canada; 4 Department of Biomedical Engineering, McGill University, Montreal, Quebec, H3A 2B4 Canada; 5 Department of Bioengineering, McGill University, Montreal, Quebec, H3A 0C3 Canada

## Abstract

Human tumour progression is a dynamic process involving diverse biological and biochemical events such as genetic mutation and selection in addition to physical, chemical, and mechanical events occurring between cells and the tumour microenvironment. Using 3D bioprinting we have developed a method to embed MDA-MB-231 triple negative breast cancer cells, and IMR-90 fibroblast cells, within a cross-linked alginate/gelatin matrix at specific initial locations relative to each other. After 7 days of co-culture the MDA-MB-231 cells begin to form multicellular tumour spheroids (MCTS) that increase in size and frequency over time. After ~15 days the IMR-90 stromal fibroblast cells migrate through a non-cellularized region of the hydrogel matrix and infiltrate the MDA-MB-231 spheroids creating mixed MDA-MB-231/IMR-90 MCTS. This study provides a proof-of-concept that biomimetic *in vitro* tissue co-culture models bioprinted with both breast cancer cells and fibroblasts will result in MCTS that can be maintained for durations of several weeks.

## Introduction

Breast cancer patients with endocrine receptor-positive (ER-positive), or human epidermal growth factor receptor-2-positive (HER2-positive), tumours are eligible for treatment with therapies targeted against these markers. However, patients with tumours that do not express ER, progesterone receptor (PR), or HER2 markers represent about 15% of patients and form the triple negative (TN) subclass, associated with poor survival and increased recurrence^1–3^. We now understand that tumours are heterogeneous and that the tumour microenvironment plays key roles in tumour evolution and resistance to therapy^4, 5^. Solid tumour growth *in vivo* occurs in a three-dimensional (3D) environment with cells in constant, and intimate, contact among the extracellular matrix (ECM) and stromal cells such as fibroblasts and macrophages^6, 7^. In the tumour microenvironment cancer associated fibroblasts (CAFs) are known to have multiple key signaling roles in tumour progression and metastasis^8, 9^.

To accurately determine the magnitude of the influence CAFs contribute in these roles the precise control over the localization, cell density, and matrix biochemistry of the stromal cells and tumour epithelial cells need to be highly controlled. 3D cell culture, co-culture of cancer cells, and cancer associated cells, grown in polymeric matrices have been shown to more accurately represent the physiological environment of tumours due to the cell-cell and cell-matrix interactions that can occur^10–12^. A variety of fabrication methods including photolithography, soft lithography, microstamping, and bioprinting have been developed to create 3D culture models^13–16^. Bioprinting is advantageous in that more complex geometric matrices can be printed with high cell density and viability and cell-laden samples can be created directly, with precise reproducibility, from cell-hydrogel suspensions^16–23^. Recently, ejection bioprinted ovarian cancer co-culture models including CAFs demonstrated that the ovarian cells were able to proliferate and spontaneously form multicellular acini^24^.

Here we report the ability of an extrusion bioprintable composite hydrogel formulation composed of ionically cross-linked alginate and gelatin hydrogels drives the formation of multicellular tumour spheroids (MCTS) without the use of additional chemical, biological, or physical stresses. Published work from Gordon G.W.^25^, Yingjun W.^26^ and Wei S.^23^ have empirically established the high printability and biocompatibility of alginate/gelatin composite within the concentration of alginate between 3–4 w/v% and gelatin of 7–8 w/v%. The material is mechanically tunable, and can be rapidly cross-linked upon extrusion to form a stiff shell, while forming a more loosely cross-linked core allowing cell migration in 3D. Using Multi-cartridge extrusion bioprinting allows us to develop cellularly heterogeneous samples comprised of both TN breast cancer cells and fibroblasts in specific initial locations with controlled density. The development of MCTS is quantitatively analyzed during 30-day culture periods by monitoring the MCTS surface area, frequency, and cell viability.

## Results

### Cellular heterogeneous *in vitro* model design

**Figure 1:**
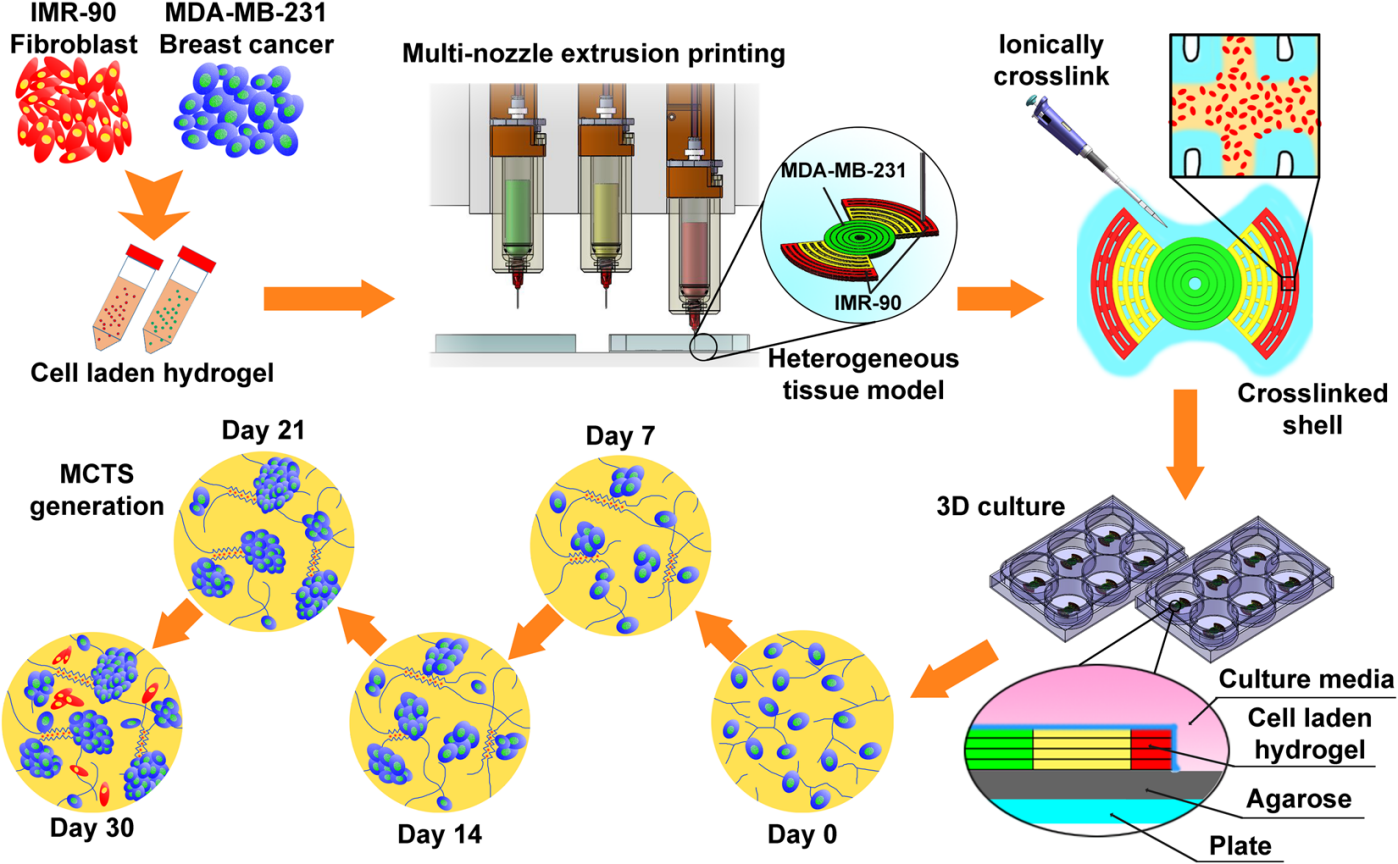
Schematic depicting the design, and experimental protocol, used to create a heterogeneous tumour model comprised of both MDA-MB-231 triple negative breast cancer cells and IMR-90 fibroblasts.

Our models were designed to incorporate both IMR-90 fibroblast cells (CAFs, cytoplasmic mCherry labeled), and MDA-MB-231 (nuclear GFP-labeled) breast cancer cells, suspended within a bioprintable cell-laden hydrogel matrix. The cells were mixed individually into a composite hydrogel solution comprised of 3% alginate/7% gelatin (w/v%). The cell-laden hydrogel solution is then gelled, and extruded to create a design consisting of a central hub of MDA-MB-231 cells adjacent to a hydrogel region of predefined dimensions that does not contain cells, and flanked by an outer segment of IMR-90 containing hydrogel. The distance between the cancer and fibroblast cells can be defined and in our proof-of-concept experiments we calculated the distance to enable the printing of equivalent numbers of each cell type as well as the volume of material deposited. The design was also selected to show that this method can produce samples directly into conventional cell culture supplies such as standard 6-well plates. Agarose is coated at the bottom of plates to minimize cell adhesion, or migration, out of the hydrogel during long-term culture periods (Fig. 1).

### Rheological properties of the composite hydrogel

The mechanical characteristics of the alginate/gelatin matrix were analyzed using rheometry and analyzing temperature sweep, time sweep, thixotropy and flow curves.

#### Temperature sweep

Using a temperature ramp from 25 °C to 37 °C at a rate of 0.2 °C/min to allow thermal stabilization under the geometry we found a decreasing storage modulus (G’) from 468.5 ± 34.2 Pa to 3.2 ± 0.2 Pa, and the loss modulus (G”) initially 140.7 ± 9.3 Pa at 37 °C decreasing to 11.8 ± 0.6 Pa at 25 °C (Fig. S1a). This reduction in G’ and G” values correspond to a denaturing of the secondary structure of the gelatin fibers resulting in a more liquid-like behavior of the material^27^. The hydrogels transition point occurs at 30.6 °C where G’ = G” = 51.7 ± 9.7 Pa enabling the hydrogels to be mixed with cells at 32 °C.

#### Gelation time sweep

**Figure 2:**
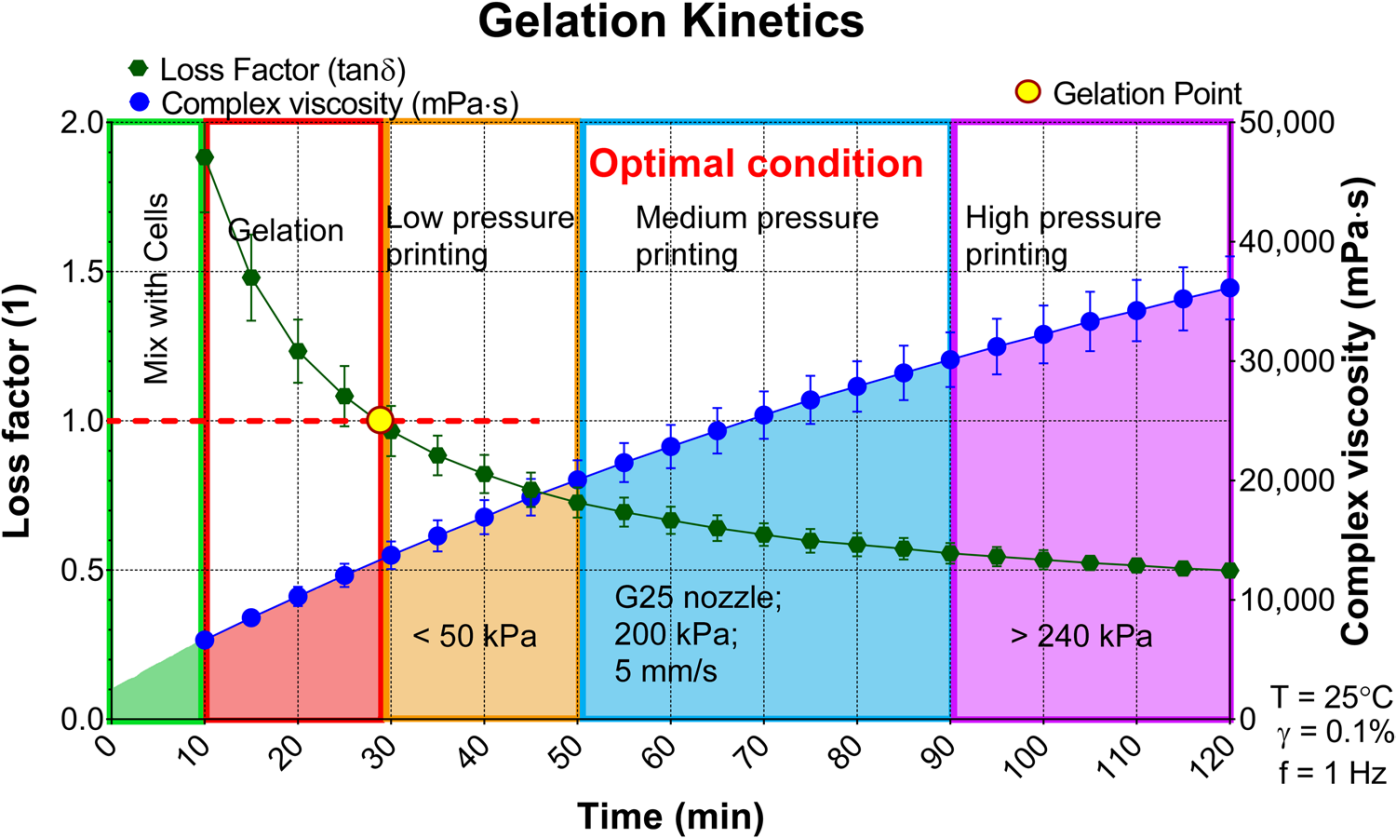
Gelation kinetics of the 3% alginate/7% gelatin hydrogel composite. The green spheres and fit represent the loss factor (left y-axis), the blue spheres and fit represent the complex viscosity (right y-axis). The red dashed line shows where the loss factor equals one indicating the gelation point of the hydrogel. Results showed in mean ± SD, n = 3.

To analyze the gelation kinetics of the alginate/gelatin matrix we implemented a time sweep where the hydrogel was removed from a 32 °C water bath and placed directly onto the rheometer platform that was heated to 25 °C. A 1 Hz frequency, 0.1% strain, was applied based on data derived from a prior amplitude sweep measurement. The initially high loss factor shows a rapid decrease occurring within the first 30 min while an increase in the complex viscosity occurs (Fig. 2). The sol-gel transition happens at approximately 30 min followed by a continuous rise in viscosity until the material reaches a period of optimal printing characteristics that occurs between 50 min to 90 min. Based upon the gelation kinetics results the cells can be mixed within the hydrogel matrix during the first 10 min and extrude under optimal conditions using 200 kPa pressure, 5 mm/s print speed, through a G25 gauge cylindrical nozzle. Notably, the hydrogel was printable between 30 min to 50 min with a lower pressure (<50 kPa) yet it was too weak to maintain structural integrity post-extrusion. 90 min after preparing the cell-laden hydrogels high pressure (>240 kPa) are required, due to increasing gel stiffness over time, which may led to decreased cell viability due to high shear stress induced cell membrane damage^21^.

#### Thixotropy tests

To simulate the extrusion process an isothermal time sweep including two independent external shear applications simulating first, the gel mixing conditions, secondly the printing process. We found an increase of both the storage and loss modulii from 11.5 ± 0.2 Pa and 27.6 ± 0.2 Pa (Fig. S1b) occurs. At 10 min a shear rate of 15 s^−1^ was applied for 1 min to simulate the mixing process, and the results show that the gentle mixing does not affect gelation kinetics aside from a minor dynamic viscosity decrease from 5,887.4 ± 139.4 mPa·s to 5,677.8 ± 128.2 mPa·s (Fig. S1b). The storage modulus curve intersected that of the loss modulus 24 min after mixing with cells into the hydrogel indicating that the material had undergone gelation. A 100 s^−1^ shear rate was performed after 41 min resulting in a significant decrease in both the storage and loss modulus from 73.5 ± 2.7 Pa and 68.3 ± 1.7 Pa to 51.6 ± 0.4 Pa and 57.2 ± 1.0 Pa, respectively, while the dynamic viscosity decreased from 3,245.2 ± 42.1 mPa·s to 2,613.9 ± 75.9 mPa·s (Fig. S1b). Regardless of the high shear, the broken hydrogel rapidly regels within 4 min ensuring stability of the bioprinted structure. Post-printing the moduli continuously increases, reaching 88.3 ± 0.4 Pa and 69.5 ± 1.00 Pa for storage and loss modulus, before the post-crosslinking process occurs.

#### Flow curve test

Flow curve tests were performed to understand changes to the hydrogels viscosity under varied shear rates. At low shear rate regimes the material exhibits a viscosity of 5.1 ± 0.2 Pa·s that decreases as the shear rate is increased, reaching 1.1 Pa·s at 100 s^−1^ (Fig. S1c). The inverse relationship indicates the composite hydrogel material is shear thinning.

### Hydrogel composition characterization

Following mechanical characterization of the hydrogel, physico-chemical analysis including FT-IR, ^13^C-NMR and SEM were performed (Fig. S2). FT-IR analysis of the alginate/gelatin hydrogel (Fig. S2 and Table S1) reveals expected polysaccharide and carboxylate bands for alginate (Fig. S2aII and Table S1)^28, 29^, as well as amide I and III bands of gelatin (Fig. S2 and Table S1)^30, 31^. ^13^C-NMR confirms the presence of gelatin (10–60 ppm)^32^, as well as alginate (65–110 ppm) (Fig. S2 and Table S1)^33^. Qualitative morphological analysis via SEM shows significant porosity throughout the material on the micron scale, which may led to enhanced cell growth due to an increased surface area, and greater exchange rates of essential nutrients and gases (Fig. S2).

### MCTS development within bioprinted 3D environments

For the material to be capable of creating the bioprinted *in vitro* models containing two, or more, different cell types the following critical parameters must be meet: (1) the gel has to be capable of handling when in a liquid form in order to obtain a homogeneous dispersion of cells; (2) the gelation temperature and gelation kinetics allow rapid gelation to minimize the exposure of the cells to room temperature conditions. To create the cell-laden hydrogel suspensions we first incubate the hydrogel at 32 °C before mixing with the chosen cell line and then temporarily lower the temperature to induce gelation.

Previously, 3D *in vitro* culture systems have been widely used to produce MCTS models with a number of different cancer cell types^7, 34^. The typical doubling time of cells within 3D cultures is approximately 6 days^35^, thus to optimize our long-term experiments we cultured the MDA-MB-231-GFP cells within the hydrogels at different initial concentrations over 30 days to determine the optimal cell concentrations within the gels. Since the MCTS display different morphologies, and not necessarily a perfect spherical shape^34, 36^, we used the surface area as a measure of MCTS growth and classified the MCTS into three distinct categories: small (500–10,000 µm^2^), medium (10,000–20,000 µm^2^), and large (>20,000 µm^2^).

By using an initial concentration of 1 × 10^6^ MDA-MB-231 cells/mL of gel we are able to induce small MCTS development after 7 days in culture. The quantities and sizes of the MCTS increase over time until reaching large MCTS sizes (>20,000 µm^2^) at ~21 days (Fig. S3a–d,q). By increasing the initial MDA-MB-231 cell concentration to 2 × 10^6^ cell/mL we observe large MCTS at both 15 and 30 days (Fig. S3e–h,r), without any large or medium MCTS occurring at 21 days, suggesting a dissociation of the medium and large MCTS from the gel, or migration out of the hydrogel into the surrounding media^37^. Increasing the MDA-MB-231 concentration to either 4 × 10^6^ or 10 × 10^6^ cells/mL results principally in small MCTS suggesting that the concentration of cells inhibits the formation of the larger MCTS sizes (Fig. S3i–l,s for 4 × 10^6^ and Fig. S3m–p, t for 10 × 10^6^ cells/mL). The potential mechanism behind the dissociation and inhibition of larger MCTS sizes, when high initial cell concentrations are employed, may be due to contact inhibition, or inhibition of the integrin β1 subunit^37, 38^. Alternatively, the cells may be creating, and reorganizing, their own matrix during these extended culture periods that is more conducive to cell adhesion retarding the formation of the larger MCTS sizes^39^.

### Cellular behavior within heterogeneous 3D models

**Figure 3:**
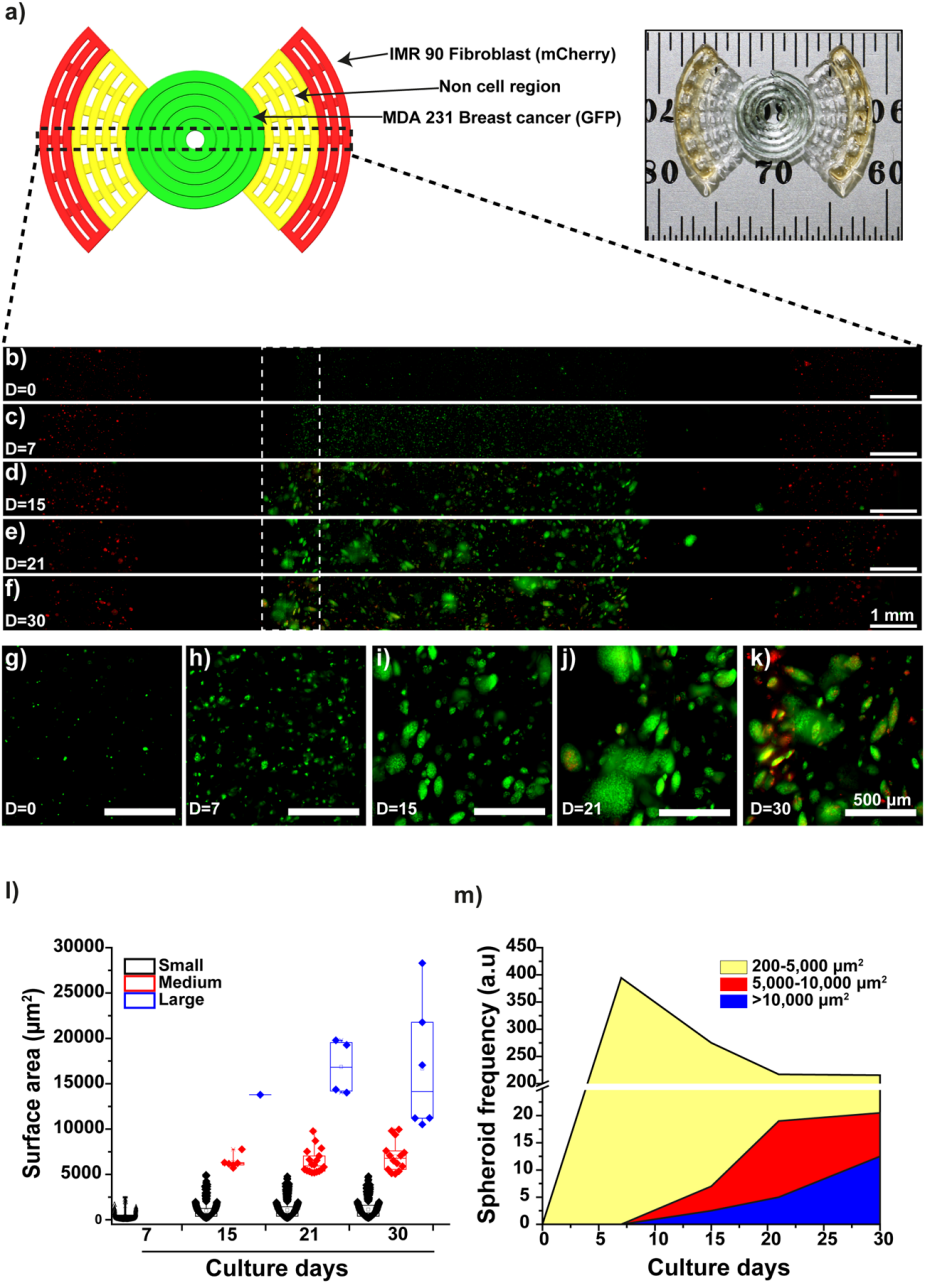
MCTS formation within a 3D bioprinted *in vitro* model consisting of IMR-90 fibroblasts and MDA-MB-231 triple-negative breast cancer cells. (**a**) CAD model and photograph of the bioprinted *in vitro* sample. (**b**) Confocal time-lapse image of MDA-MB-231-GFP (green) and IMR-90-mCherry (red) cells bioprinted within the model (**b–f**) and their zoom-in (**g**–**k**). Scale bar is 1 mm (**b**–**f**) and 500 µm for selected areas (dotted line (**g**–**h**)), magnification x10. (**l**) MCTS formation and size quantification during a 30 day period: 500–10,000, 10,000–20,000 and >20,000 µm^2^ for small, medium and large spheroids, respectively. (**m**) Frequency of MCTS distribution as a function of time cultured. Box plot graphs were plotted using a box limit of 25^th^ and 75^th^ percentiles and a minimum-maximum whisker’s range.

Due to favorable growth kinetics, the heterogeneous cell-laden *in vitro* bioprinted models used an initial cell concentration of 1 × 10^6^ MDA-MB-231 cells/mL suspended within the gel (Figs 3a and S4). After 7 days of culture the MDA-MB-231 cells again formed small MCTS (Fig. 3c,h) with a similar trend of increasing MCTS size over time (Fig. 3d–f,i–k). At day 15 medium and large MCTS (Fig. 3l) were observed with increasing frequency while the frequency of the small MCTS decreased suggesting a proliferation of individual cells within the MCTS (Fig. 3m). After 30 days the largest MCTS within the gels achieved a surface area up to 80,000 µm^2^. The IMR-90 cells printed within the model began to migrate through the non-cell hydrogel region and infiltrate the MDA-MB-231 cells after ~15 days and continued through the remaining culture period creating mixed MDA-MB-231/IMR-90 MCTS.

**Figure 4:**
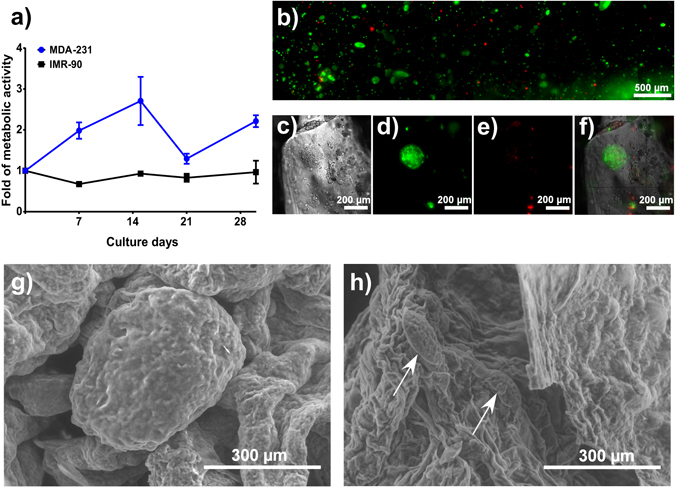
Cell viability and MCTS development. (**a**) Cell viability of MDA-MB-231-GFP and mCherry-IMR-90 during 30 days of culture within the hydrogel. (**b**) LIVE/DEAD assay of unlabeled MDA-MB-231 cells after 21 days of culture with a representative full view (**b**) across the width of the entire sample. (**c**) Magnified bright field image of a representative MCTS, (**d**) fluorescence image of live cells stained with the LIVE/DEAD assay, (**e**) fluorescence image of dead cells stained with the LIVE/DEAD assay, (**f**) merged bright field and fluorescent LIVE/DEAD assay images, (**g**–**h**) SEM images of MDA-MB-231 MCTS within the gel after 21 days of culture showing (**g**) medium and (**h**) small MCTS (indicated with pointed arrows) under a magnification of 150x. Results showed in mean ± SD, n = 3.

While an increase of MCTS size is one index of cell proliferation within the bioprinted model we also monitored metabolic activity and cell permeability to evaluate cell health, proliferation, and MCTS generation. MTS and LIVE/DEAD assays are ones of the most common methods used to evaluate the viability of cells. The reduction of tetrazolium salts in the MTS assay is driven by mitochondrial dehydrogenases, where the quantity of formazan products measured is directly proportional to the number of viable cells. On the other hand, LIVE/DEAD assay uses two different compounds, one is nonfluorescent cell-permeant calcein-AM which is degraded by intracellular esterases producing an intense green fluorescence in living cells, and the second compound is ethidum homodimer (EthD-1) which penetrates cells with damaged membrane producing bright red fluorescence in dead cells without entering to live cells. The metabolic activity of IMR-90 cells remains constant over time (Fig. 4a) while the metabolic activity of the MDA-MB-231-GFP cells increased during the first 15 days, then decreased at day 21, and increases again at day 30. This is likely due to the metabolic activity that occurs in the medium, and large MCTS, compared to the small MCTS as well as single cells proliferating within the matrix^40, 41^.

To further evaluate our bioprinted *in vitro* tumour model cell viability was quantified using a LIVE/DEAD assay on non-labeled MDA-MB-231 with an initial cell density of 1 × 10^6^ cells/mL. A significant number of the MCTS (Fig. 4b) appear viable although a necrotic core occurs dependent upon MCTS size (Fig. 4c–f), confirming the result obtained with MTS assay. The structure of the MCTS inside the gel was visualized using environmental Scanning Electron Microscopy (eSEM) after growing in culture for 21 days. Medium (Fig. 4g) and small (Fig. 4h, indicated with pointed arrows) MCTS were distributed within the samples, and the cell printed models at this point differ significantly from both SEM images of the hydrogel without cells (Fig. S2c,d).

Conventional models of breast cancer have challenges in the formation and study of physiological mimicking MCTS due to their lack of spatial cellular heterogeneity and dependence on external stimuli or stresses that may influence cell function or behavior. 3D bioprinting technology overcomes some of the challenges by enabling the fabrication of heterogeneous *in vitro* models in highly biocompatible hydrogels with predetermined initial locations and concentrations of both cancer cells and cancer-associated cells.

## Discussion

Alginate and gelatin are used as a biocompatible composite hydrogel bioink to embed, and extrude, breast cancer cells and CAFs into preprogrammed initial locations. The alginate component of the gel imparts viscous properties during printing, and is ionically cross-linked post-extrusion to provide mechanical reinforcement, while the gelatin component provides elastic characteristics in addition to bioactive moieties that promote cell adhesion. These composite hydrogels have tunable mechanical properties including their shear moduli, loss factor, and complex viscosity that can be modulated to achieve the desired stiffness.

The current methods to produce MCTS force single cells to form aggregates by physical confinement such as the hanging drop method^42^, or chemical induction by the addition of peptides^43^, which can alter cell physiology and biochemistry. The composite hydrogels creates a biomimetic environment that facilitates MCTS formation without the need for external stressors.

These models can provide significant improvements to alternative 3D cell culture models by enabling long culture periods (>30 days) of more than one cell type within a biomimetic environment with predictable outcomes such as the frequency, size, and cellular composition of MCTS. These models provide insights into reconstructing physiological-mimicking *in vitro* tissue models with cellular heterogeneity that can enable insight into cell-cell interactions, diagnostic and therapeutic discovery, and tumourigenesis mechanisms.

## Methods

### Material preparation

Sodium alginate (Protanal LF 10/60 FT, FMC BioPolymer) and Type B gelatin from bovine skin (G9391, Sigma-Aldrich) powders were sterilized via UV exposure overnight. The powders were then dissolved in DPBS (1×, w/o calcium, w/o magnesium, Gibco) and stirred using a magnetic hotplate for 1 hour at 60 °C and 2 hours at room temperature to achieve a homogeneous composite precursor comprised of 3 w/v% alginate and 7 w/v% gelatin. The composite precursor was transferred to centrifuge tubes and centrifuged at 2,000 rpm for 5 min to eliminate bubbles. It was then stored in 4 °C fridge and used within one week. A 100 mM CaCl_2_ solution for crosslinking the alginate was also prepared by dissolving CaCl_2_ (Sigma-Aldrich) into MilliQ water and stored in 4 °C fridge until use. All material preparation for biological testing was carried out under sterile conditions all the time.

### Mechanical tests

Mechanical properties were characterized by rheometry. The data was collected using an oscillation rheometer MCR 302 (Anton Paar). Thin disks of materials with dimensions Φ25 mm × 1 mm were prepared for analysis. Parallel measuring geometry with diameter of 25 mm (PP25, Anton Paar,) was mounted to the rheometer. All the experiments were performed in triplicate.

An amplitude sweep was first implemented to find the linear viscoelastic range. A strain sweep from 0.01% to 100% was applied to the gel at a frequency of 0.01 Hz and 10 Hz. A temperature ramp was carried out from 25 °C to 37 °C at a rate of 0.2 °C/min to allow thermal stabilization at 0.1% strain. In the time sweep analysis the hydrogel sample was taken from a 32 °C water bath and placed directly onto the rheometer platform that was heated to 25 °C. A 1 Hz frequency 0.1% strain was applied based on data derived from a prior amplitude sweep.

A simulation of the extrusion process using an isothermal time sweep with two intersections where external shear was applied to simulate both the mixing and printing process was performed where after 10 min of the gelation process a shear rate of 15 s^−1^ was applied for 1 min to simulate the mixing process followed by the application of a 100 s^−1^ shear rate at 41 min to simulate the extrusion process. The applied shear rate was approximated via using the equation: $\dot{\gamma }=\frac{4V}{R}$, where *V* is the average flow velocity and *R* is the radius of nozzle.

### Physico-chemical characterization

To confirm the presence of alginate and gelatin in our hydrogel physico-chemical experiments including FT-IR and NMR spectroscopy were performed. For FT-IR experiments hydrogels were freeze-dried overnigth and the powder was analyzed using a FTIR-ATR spectrophotometer (Nicolet 6700/Smart iTR, Thermo Scientific); the results were plotted in a wavelength range from 4000 to 500 cm^−1^. The chemical structure of alginate and gelatin was further analyzed by ^13^C-NMR. Lyophilized hydrogel was dissolved into D_2_O at a concentration of 50 mg/mL and NMR experiments were performed in a Bruker Avance 600 spectrometer (NMR 600 MHz, Avance III HD, Bruker) at 40 °C, with 20,000 scans (17 h acquisition) and a delay adopted of 2 s.

### SEM imaging

To study the internal structure and morphology scanning electron microscopy (SEM, Hitachi SU-3500 Variable Pressure) of cross-linked hydrogels previously frozen in liquid nitrogen was performed. The cells in hydrogels were fixed by immersing in 4% paraformaldehyde for 30 min at 37 °C, then rinsed with DPBS, rapidly frozen in liquid nitrogen for 1 min, and freeze-dried the samples overnight prior to imaging at 25.0 kV and 70 Pa at magnification of 40x up to 5,000x.

### Cell preparation

For *in vitro* experiments MDA-MB-231 and IMR-90 cell lines transfected with GFP (nuclear expression) and mCherry (cytoplasmatic expression), respectively, were cultured at 5% CO_2_, 37 °C in DMEM medium (Gibco) at pH 7.2 supplemented with 10% fetal bovine serum (Wisent Bioproducts), 100 U/mL penicillin, 100 *μ*g/mL streptomycin, and 0.25 *μ*g/mL, and amphotericin B (Sigma) in T-75 flasks (Corning). After three passages when 80% of cellular confluence was reached, the cells were rinsed twice with DPBS and then harvested with trypsin-EDTA (0.25%-1X, Gibco).

### Scaffold design and 3D fabrication

We designed a propeller-like model where breast cancer cells (MDA-MB-231) were placed in the center of the model and CAF cells (IMR-90) at the edges with a non-cell containing zone initially separating both types of cells. The CAD model was created using SolidWorks software (Dassault Systems) and converted to an STL file before being imported into Sli3r (open source software) to generate standard G-Code. A MATLAB (The MathWorks Inc) script was written to convert the standard G-code to the specific G-code used by the 3D printer. The propeller model has an internal circle with diameter 7.7 mm, and external parts comprised of two sectors with maximum radius of 8.65 mm (Fig. 3). The parameters were calculated to ensure the areas of both cell-laden regions were identical. Each of the propeller models comprised of 4 interlaced layers with layer thickness of 150 µm. The adjacent hydrogel lines had a gap of approximately 500 µm to allow exchange of nutrients and gas.

The samples were printed using a 3D bioprinter BioScaffolder 3.1 (GeSiM). The printer features a three axes platform with XY resolution of 2 µm and Z resolution of 10 µm. Three extrusion cartridges were installed onto the printing head and driven by a pneumatic system. The controlled pressure pushes the piston within the cartridge and extrudes the material through a dispensing nozzle onto the platform.

Prior to printing, the 3% alginate plus 7% gelatin hydrogels were transferred to a 32 °C water bath for 1 h to reach thermal equilibrium. Upon printing, the hydrogels were divided into three cartridges and loaded with breast cancer cells, fibroblast cells, and non-cell containing gels respectively. This was performed during the first 10 min after removing the samples from the 32 °C water bath to mix cells while the samples are in a liquid form to allow homogeneous distribution. The three cell-laden/non-cell cartridges were kept at 25 °C for 50 min to reach the optimal printing conditions (Fig. 2). During printing the cell-laden hydrogels experience an increased shear rate during extrusion and consequently a decrease in integrity. When forming models on the platform the integrity rebounds and holds the structure. The printing head travels along a preprogrammed trajectory and fabricates the scaffold layer by layer. After printing all replicates of the propeller models excessive 100 mM CaCl_2_ cross-linking solution was added to the models for 1 min before rinsing by DPBS which rapidly cross-links the alginate component within the models. The cell-laden propeller models were then cultured in 6 well plates in an incubator at 37 °C and 5% CO_2_.

### Viability and spheroid formation experiments

To determinate the best cell concentration for long-term *in vitro* culture, MDA-MB-231 with an initial concentration of 1, 2, 4 and 10 × 10^6^ cells/mL were mixed into 100 µL hydrogel disks cross-linked with CaCl_2_ for 1 min at room temperature and transferred into agarose-coated 6-well plates; then, the disks were cultured at 37 °C with 5% CO_2_ for 30 days. The culture media was replaced every 3 days. We analyzed the spheroid size and shape at 0, 7, 15, 21 and 30 days using a confocal spinning disk inverted microscope (Olympus IX83, Olympus Life Science). Images were acquired at multiple positions and z-stacks were acquired and reconstructed using a maximum stack arithmetic tool to create the 2D images for spheroids analysis. We used an MTS assay to determine cell viability as per the manufacturer’s procedure (Promega) with some modifications. Each disk was washed, cut in 4 parts and the contents of the whole disk were transferred into a 96-well plate. Then, 100 µL DMEM plus 20 µL MTS reagent were added to each disk and incubated at 37 °C for 2 h. After the reaction the supernatant was recovered and transferred into clean 96-well plate. Samples were measured at 490 nm. As a secondary viability test a LIVE/DEAD assay (Molecular Probes, ThermoFisher Scientific) was used to analyze non-fluorescently labeled MDA-MB-231 cells using the standard protocols from the provider.

### Statistical Analysis

All experiments were replicated at least three times. Statistical analysis were processed using the built-in functions of Prism 7. Data is shown as Mean ± SD. Where comparisons are done, one-way ANOVA is used, and P < 0.05 is considered as significant. Box plot graphs were plotted using OriginPro 9 software with a box limit of 25^th^ and 75^th^ percentiles and a minimum-maximum whisker’s range.

## Electronic supplementary material

Supplementary Info
