# Supplementary material for: Directing the Self-assembly of Tumour Spheroids by Bioprinting Cellular Heterogeneous Models within Alginate/Gelatin Hydrogels
Source: Sci Rep. 2017 Jul 4;7:4575. doi: 10.1038/s41598-017-04691-9 (PMC5496969; doi:10.1038/s41598-017-04691-9)
Supplement: Supplementary file 1 — Supplementary Info [file 41598_2017_4691_MOESM1_ESM.doc]

Supplementary Information

Directing the Self-assembly of Tumor Spheroids by Bioprinting Cellular Heterogeneous Models with Alginate/Gelatin Hydrogels

*Tao Jiang, Jose G. Munguia-Lopez, Salvador Flores Torres, Joel Grant, Sanahan Vijayakumar, Antonio De Leon-Rodriguez, Joseph M. Kinsella^*^*

[*joseph.kinsella@mcgill.ca](mailto:*joseph.kinsella@mcgill.ca)

This supporting document includes images of additional mechanical tests of the composite hydrogel as mentioned in the main context, physical characterization of the composite hydrogel, MCTS genesis analysis in 3D hydrogel with quantified data based on spheroid size, separated confocal imaging of the “propeller-like” heterogeneous 3D model, and a table showing the detailed data of FT-IR and NMR tests of the composite hydrogel.


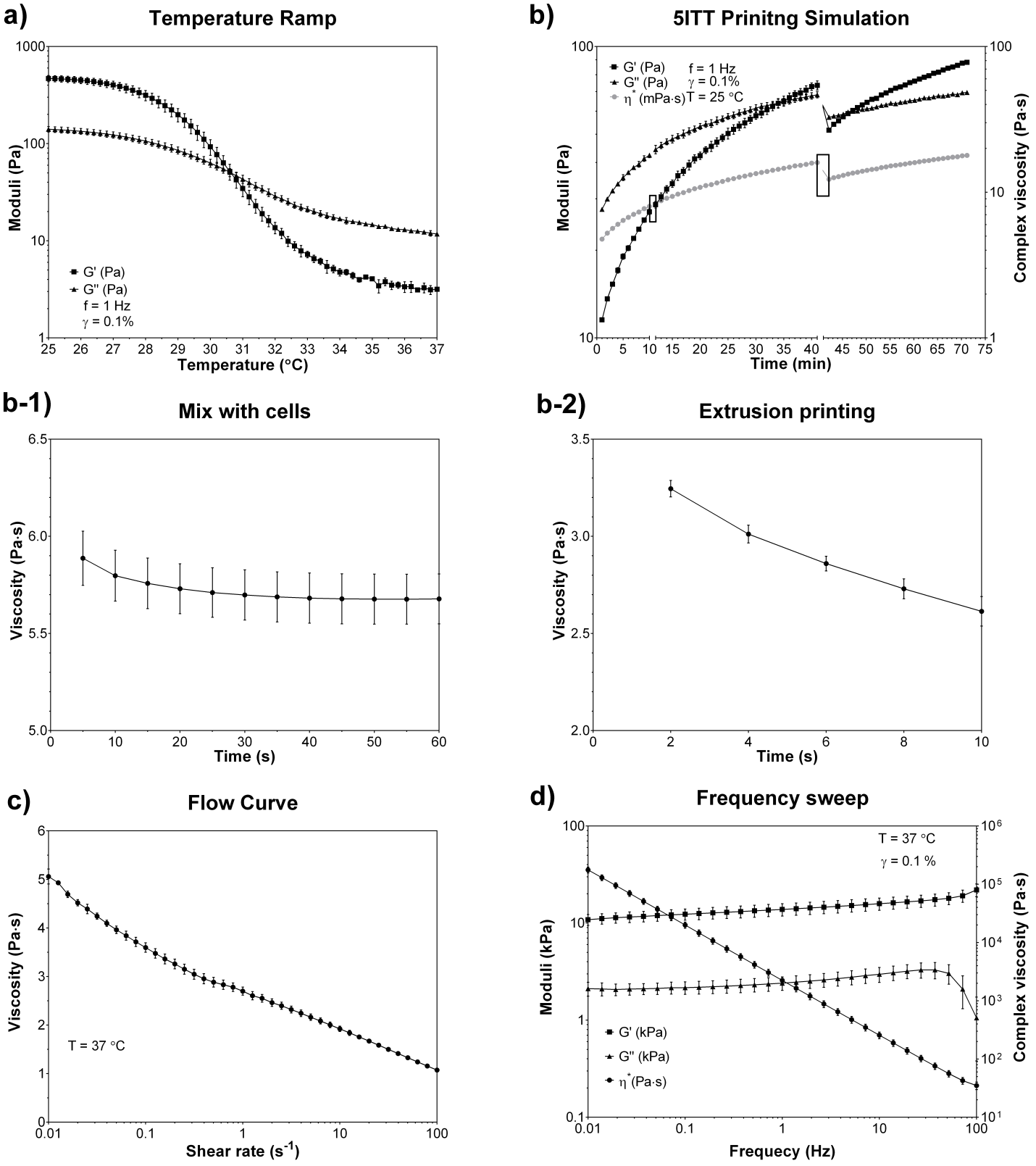


**Figure S1.** Mechanical properties of hydrogel (3% alginate 7% gelatin blend).a) Temperature ramp of the blend, a transition temperature of 30.6 ºC can be realized. b) 5 Interval Thixotropy Test (5ITT) was implemented to simulate the printing process, a decrease of mechanical integrity during the shearing process was observed but the material recovered rapidly. b-1) Zoom in view of the left box in figure S1-b), complex viscosity slightly decreased during the simulated cell mixing procedure. b-2) Zoom in view of the right box in figure S1-b), a notable decline of viscosity was seen during the simulated extrusion process. c) Flow curve of the uncrosslinked interior hydrogel, a shear thinning properties could be realized. d) Frequency sweep of the crosslinked exterior shell, a 10 kPa of storage shear modulus was obtained that provided essential integrity of the tissue model for a long period of 3D culture (> 30 days).


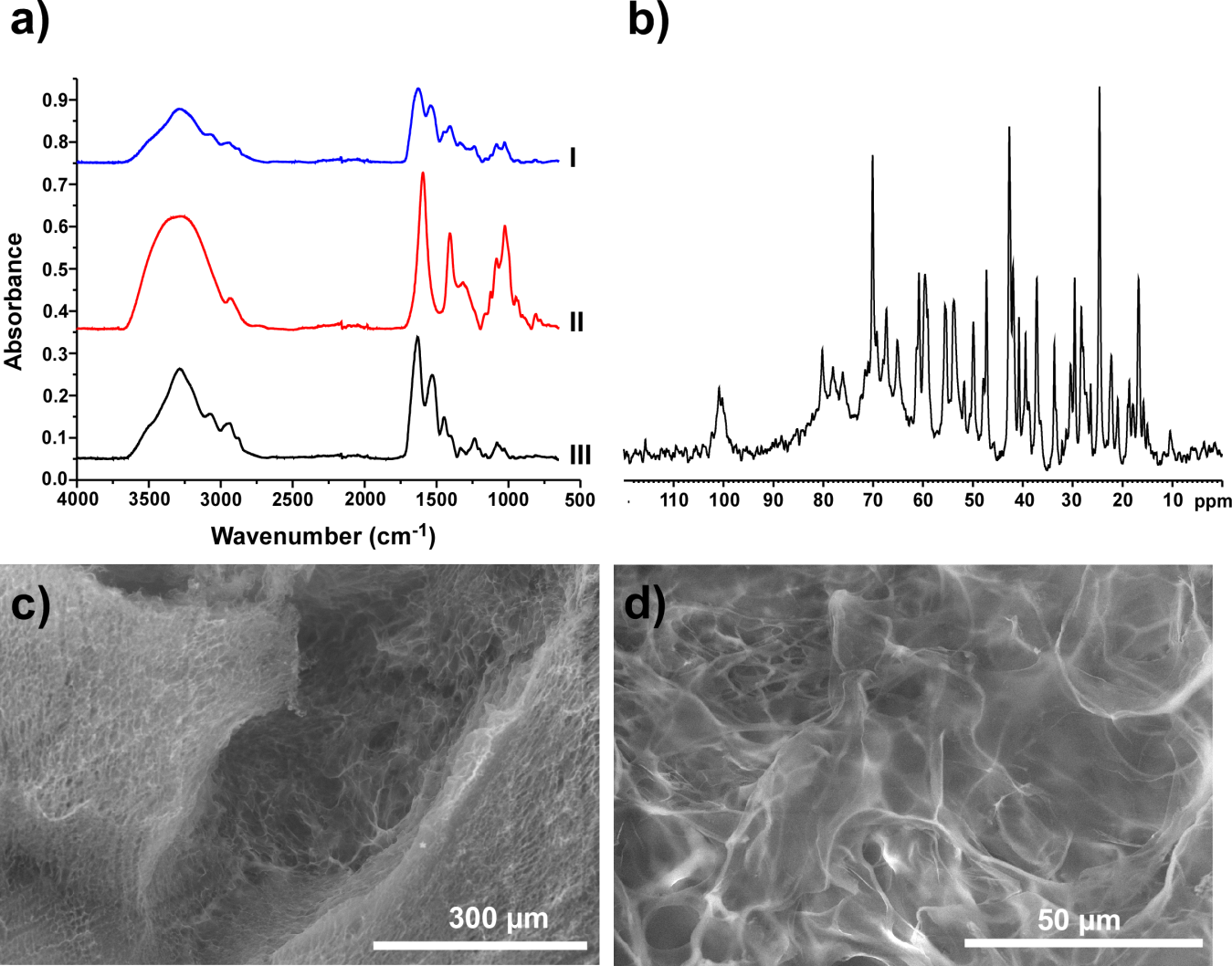


**Figure S2.** Physical characterization of hydrogel. FT-IR spectrum of alginate/gelatin hydrogel (a I), alginate (a II) and gelatin (a III). ^13^C-NMR spectra of alginate/gelatin matrix (b). SEM images of hydrogel at ×150 (c) and ×1000 (d) of magnification.


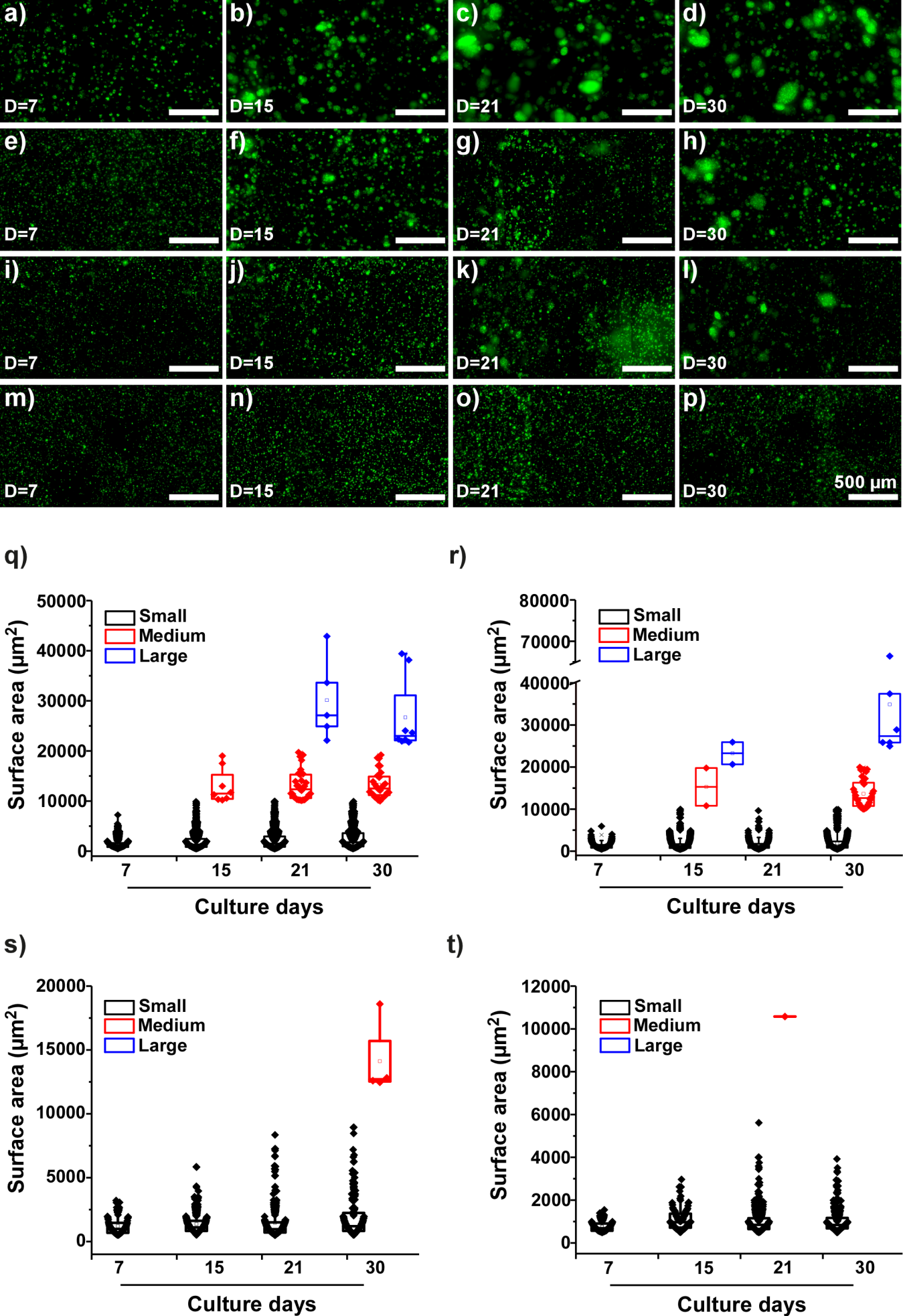


**Figure S3.** MDA-231 spheroid-like formation. Confocal image of breast cancer cells mixed with hydrogel at initial concentration of 1x10^6^ (a-d), 2x10^6^ (e-h), 4x10^6^ (i-l) and 10x10^6^ (m-p) cell per mL incubated during 30 days. Scale bar 500 µm, magnification ×10. The frequency of spheroids and their sizes where plotted in box-plot graph for 1x10^6^ (q), 2x10^6^ (r), 4x10^6^ (s), 10x10^6^ (t) cell per mL, where the threshold was 500-10,000, 10,000-20,000 and >20,000 µm^2^ for small, medium and large spheroids, respectively.


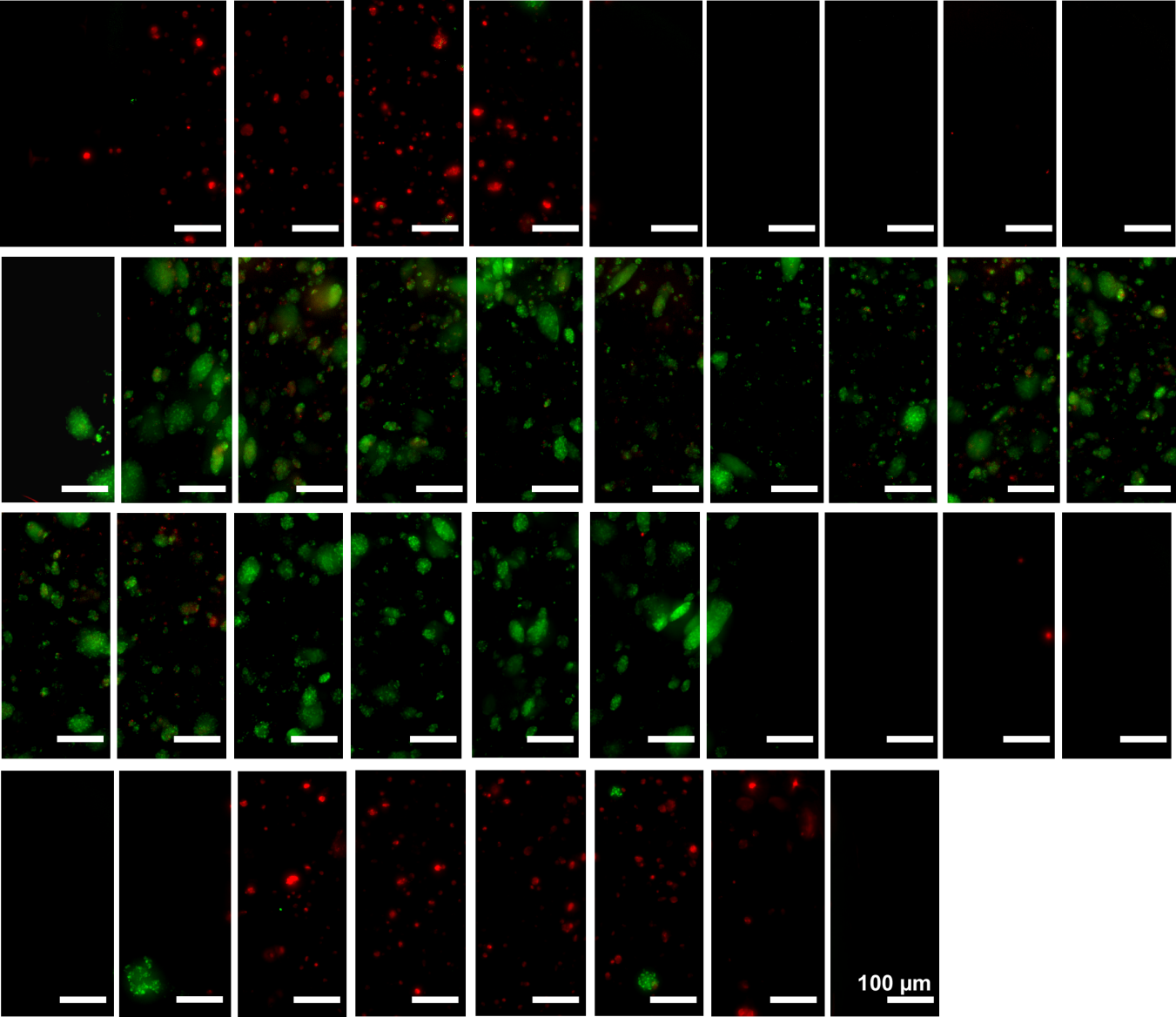


**Figure S4.** Deconstructed images of full propeller at day 15 post-culture. IMR-90 (red) and MDA-231 (green). Magnification ×10.

**Table S1.** Characteristics peaks of alginate and gelatin biopolymers.

| FTIR (wavenumber cm^-1^) | | |  | ^13^C-NMR (ppm) |
| --- | --- | --- | --- | --- |
| Alginate | Gelatin | Alginate/Gelatin |  | Alginate/Gelatin |
|  |  |  |  |  |
| 810.38 | 1080.81 | 1027.27 |  | 14.9555 |
| 947.60 | 1236.95 | 1081.99 |  | 16.6308 |
| 1024.44 | 1335.04 | 1160.49 |  | 24.5067 |
| 1082.13 | 1398.68 | 1236.41 |  | 26.3145 |
| 1123.57 | 1446.85 | 1336.33 |  | 27.7752 |
| 1317.06 | 1528.48 | 1405.28 |  | 29.5122 |
| 1406.05 | 1628.95 | 1540.40 |  | 33.5927 |
| 1594.06 | 1980.30 | 1625.10 |  | 37.0816 |
| 1980.26 | 2115.17 | 1979.96 |  | 39.3818 |
| 2161.96 | 2162.20 | 2050.18 |  | 40.6988 |
| 2931.40 | 2938.55 | 2113.17 |  | 41.8591 |
| 3253.82 | 3072.86 | 2163.13 |  | 42.5785 |
|  | 3283.01 | 2323.40 |  | 47.1832 |
|  |  | 2941.73 |  | 47.1832 |
|  |  | 3276.43 |  | 55.3134 |
|  |  |  |  | 58.9993 |
|  |  |  |  | 60.7464 |
|  |  |  |  | 67.2631 |
|  |  |  |  | 70.0376 |
